# LUCL reconstruction of the elbow: clinical midterm results based on the underlying pathogenesis

**DOI:** 10.1007/s00402-021-03759-6

**Published:** 2021-02-19

**Authors:** Stephanie Geyer, Caroline Heine, Philipp W. Winkler, Patricia M. Lutz, Andreas Lenich, Bastian Scheiderer, Andreas B. Imhoff, Sebastian Siebenlist

**Affiliations:** 1 Department for Orthopaedic Sports Medicine, Klinikum rechts der Isar, Ismaninger Str. 22, 81675 Munich, Germany; 2 Center for Elbow and Shoulder Therapy, Nymphenburgerstr. 1, 80355 Munich, Germany

**Keywords:** Elbow, Lateral ulnar ligament construction, Autologous triceps augmentation, Posterolateral instability, Pathogenesis, Functional outcome

## Abstract

**Purpose:**

Traumatic and atraumatic insufficiency of the lateral ulnar collateral ligament (LUCL) can cause posterolateral rotatory instability (PLRI) of the elbow. The influence of the underlying pathogenesis on functional outcomes remains unknown so far. The objective of this study was to determine the impact of the initial pathogenesis of PLRI on clinical outcomes after LUCL reconstruction using an ipsilateral triceps tendon autograft.

**Methods:**

Thirty-six patients were reviewed in this retrospective study. Depending on the pathogenesis patients were assigned to either group EPI (atraumatic, secondary LUCL insufficiency due to chronic epicondylopathia) or group TRAUMA (traumatic LUCL lesion). Range-of-motion (ROM) and posterolateral joint stability were evaluated preoperatively and at follow-up survey. For clinical assessment, the Mayo elbow performance (MEPS) score was used. Patient-reported outcomes (PROs) consisting of visual analogue scale (VAS) for pain, disability of arm, shoulder and hand (DASH) score, patient-rated elbow evaluation (PREE) score and subjective elbow evaluation (SEV) as well as complications were analyzed.

**Results:**

Thirty-one patients (group EPI, *n* = 17; group TRAUMA, *n* = 14), 13 men and 18 women with a mean age of 42.9 ± 11.0 were available for follow-up evaluation (57.7 ± 17.5 months). In 93.5%, posterolateral elbow stability was restored (*n* = 2 with re-instability, both group TRAUMA). No differences were seen between groups in relation to ROM. Even though group EPI (98.9 ± 3.7 points) showed better results than group TRAUMA (91.1 ± 12.6 points) (*p* = 0.034) according to MEPS, no differences were found for evaluated PROs (group A: VAS 1 ± 1.8, PREE 9.3 ± 15.7, DASH 7.7 ± 11.9, SEV 92.9 ± 8.3 vs. group B: VAS 1.9 ± 3.2, PREE 22.4 ± 26.1, DASH 16.0 ± 19.4, SEV 87.9 ± 15.4. 12.9% of patients required revision surgery.

**Conclusion:**

LUCL reconstruction using a triceps tendon autograft for the treatment of PLRI provides good to excellent clinical outcomes regardless of the underlying pathogenesis (traumatic vs. atraumatic). However, in the present case series, posterolateral re-instability tends to be higher for traumatic PLRI and patient-reported outcomes showed inferior results.

**Level of evidence:**

Therapeutic study, LEVEL III.

## Introduction

Over the last decade, lateral ulnar collateral ligament (LUCL) reconstruction has become an established procedure for chronic posterolateral rotatory instability (PLRI) management [1–8]. PLRI is primarily associated with traumatic lesion of the LUCL and was first described by O’Driscoll in 1991 [9]. Nowadays, however, a variety of atraumatic mechanisms are well known to induce secondary LUCL insufficiency with subsequent PLRI [7, 10–12]. In the absence of traumatic history, iatrogenic injuries to the lateral ligament complex during debridement of the extensor carpi radialis brevis (ERCB) or multiple corticosteroid injections due to persistent epicondylopathia humeri radialis (EPHR) are mainly reported as reasons for secondary LUCL insufficiency [10, 11, 13, 14].

LUCL insufficiency leads to lateral elbow pain and impairment in the activities of daily living. Besides clinical testing of lateral and posterolateral elbow laxity and radiographic examination (anteroposterior, lateral), MRI scans are recommended to evaluate the lateral ligament complex of the elbow and the extensor muscles as well as to exclude other causes of lateral elbow pain (e.g. loose bodies, hypertrophic humeroradial plica, osteochondral lesions). Nevertheless, in some cases, the differentiation of chronic PLRI remains challenging. Therefore, arthroscopic instability testing could be useful to determine PLRI and indicate secondary stabilization procedures [15]. In the current literature, encouraging results of LUCL reconstruction in chronic PLRI are described [14, 16–18]. Nevertheless, the effect of the triggering pathogenesis of PLRI on clinical outcomes following LUCL reconstruction is not yet differentiated.

The purpose of the present study was to determine the influence of the pathogenesis (traumatic vs. atraumatic) of PLRI on clinical outcomes after LUCL reconstruction using an ipsilateral triceps tendon autograft. It was hypothesized that LUCL reconstruction in patients suffering from atraumatic PLRI results in superior patient-reported outcomes (PROs), range-of-motion (ROM), and a lower rate of re-instability compared to patients suffering from traumatic PLRI.

## Materials and methods

### Patient characteristics

Thirty-six consecutive patients who underwent LUCL reconstruction for PLRI between November 2012 and April 2018 were included in the present study and informed consent was obtained by each patient. The local ethics committee approved the study protocol (256/19s) and the study was conducted according to the Declaration of Helsinki. The inclusion criteria contained a minimum follow-up of 24 months after surgery, a patient’s age ranging from 18 to 65 years and the LUCL reconstruction with an ipsilateral triceps graft due to a clinically and arthroscopically proven PLRI. All reconstructions were performed by two experienced elbow surgeons (A.L. and S.S.). Patients with revision LUCL reconstruction, MUCL reconstructions, concomitant dislocations fractures, nerve injuries and/or any rheumatic disease were excluded.

Based on patients’ documented history, the pathogenesis of PLRI was reviewed. Patients were subsequently assigned to group EPI (= atraumatic history) or group TRAUMA (= previous elbow injury). At follow-up survey, 31 patients (13 men, 18 women) were available for evaluation (Table 1). One patient denied clinical reevaluation for time reasons and four patients could not be traced due to unknown addresses.

**Table 1:** Demographics

	Group EPI (*n* = 17)	Group TRAUMA (*n* = 14)
Age [years, mean ± SD]	46.4 ± 7.3	38.6 ± 13.4
Sex [f/m]	10/7	8/6
BMI [kg/m^2^, mean ± SD]	25.6 ± 4,1	25.9 ± 5.5
Hand dominance, [r/l]	15/2	12/2
Beighton score [30]	1.4 ± 2.1	2.75 ± 3.0
Previous surgeries	6	5
Interval—symptoms to surgery [months, mean ± SD]	41.3 ± 56.5	61.3 ± 93.8
Follow-up, mean ± SD [months]	60.1 ± 17.4	54.9 ± 18.4

*SD* standard deviation, *f* female, *m* male, *kg* kilogram, *m* meter, *r* right, *l* left

**Fig. 1:**
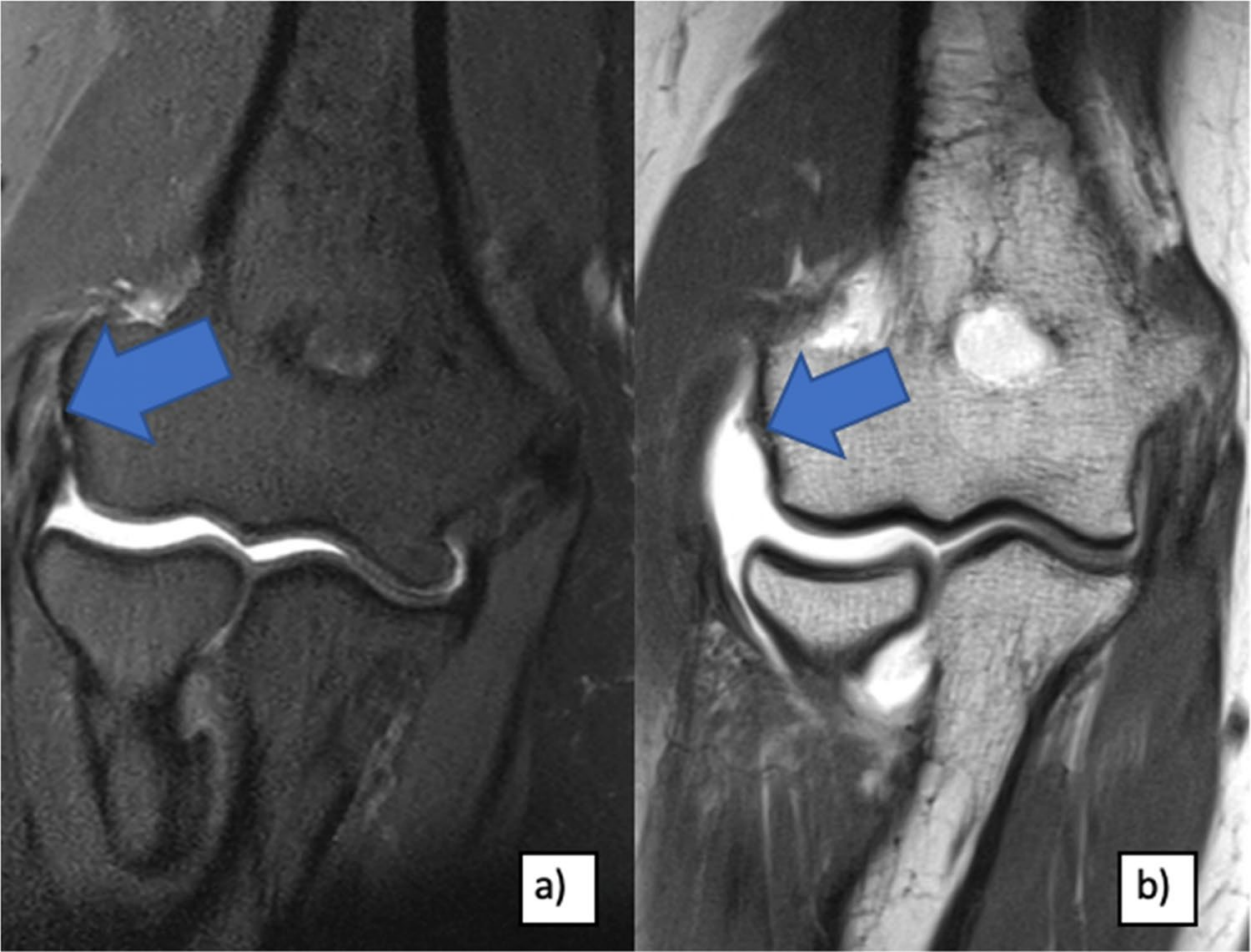
MRI of patients with atraumatic LUCL insufficiency (right elbow): **a** partial lesion of LUCL and extensor tendons (blue arrows) after multiple steroid injections and **b** complete LUCL and extensor tendon avulsion (blue arrow) following Hohmanns’ surgery

In group EPI, PLRI was figured out following previous steroid injections (*n* = 12), following extensor tendon release procedures (mostly described as “Hohmann’s surgery” [15] 19) (*n* = 3) and following steroid injections combined with extensor tendon surgery (*n* = 2) (Fig. 1). For all patients treated with corticosteroids, injections were performed 3 times on average (range, 1–30 times). One patient had undergone an additional decompression of the radial nerve accompanying to steroid injections.

The injury mechanism for PLRI in group TRAUMA was induced by elbow dislocations in four cases, by elbow subluxation (sensation of slight joint incongruence) during a high-energy trauma in four cases (car or bicycle accident), following a radial head fracture in three cases (*n* = 3 Mason type II fractures [20]) and by a fall on the out-stretched arm from a standing height in three cases. Within this trauma group, five patients underwent previous surgeries. Three patients received an osteosynthesis of the radial head. Hardware removal followed after 1 year and one patient underwent additional arthroscopic arthrolysis. In one patient with an elbow dislocation, the LCL complex with the CEO (= common extensor origins) was initially re-fixed. In one patient, an arthroscopic arthrolysis was performed due to a posttraumatic elbow stiffness following dislocation injury.

### Arthroscopic instability testing

**Fig. 2:**
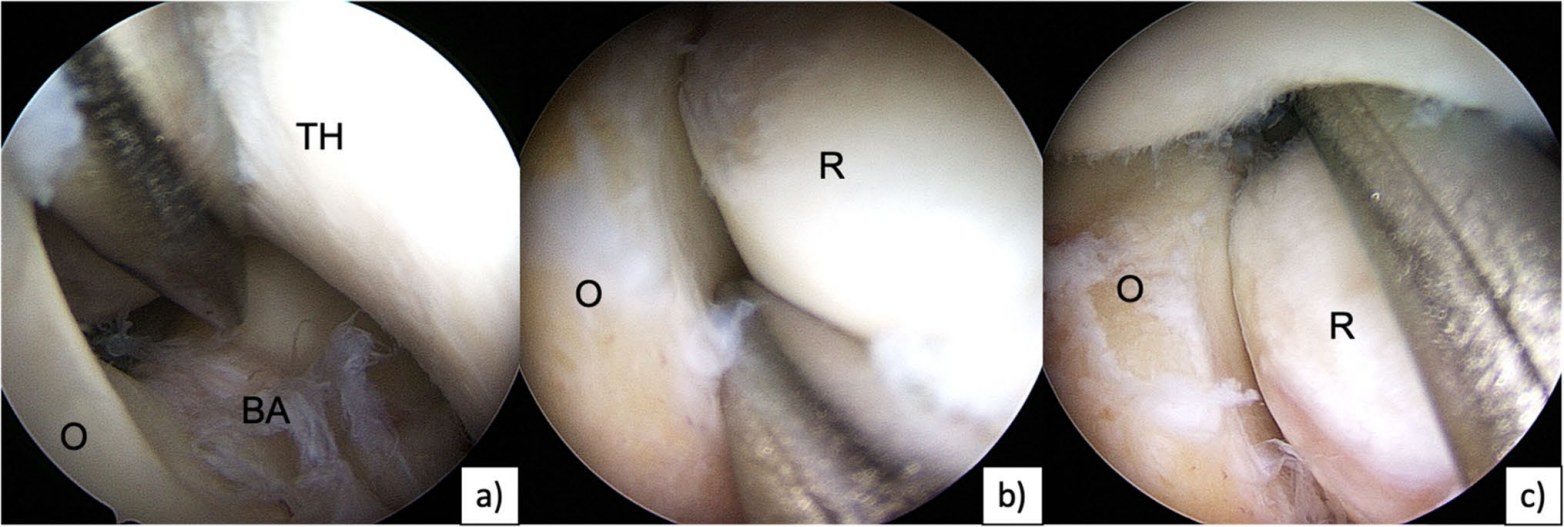
Arthroscopic instability testing with a 4 mm switching stick of a right elbow with symptomatic PLRI: **a** humeroulnar dorsal: grade III, **b** radioulnar: grade I, **c** humeroradial: drive through sign, grade III. *TH* trochlea humeri, *R* radial head, *O* olecranon, *BA* bare area

In all patients, PLRI was confirmed during arthroscopic stability testing. For arthroscopic instability testing, the classification of Geyer et al. was used [21, 22]: grade I (= stable), the switching stick (5 mm) cannot be pushed into joint spaces (posterolateral, radioulnar, humeroradial,); grade II (mild instability), the joint partners can be pushed away with the switching stick; grade III (grossly instable), the switching stick drives through into the anterior joint compartment provocating a posterolateral subluxation (Fig. 2). For grade II combined with positive clinical instability testing as well as for grade III, LUCL reconstructions were indicated.

### Surgical management of PLRI

**Fig. 3:**
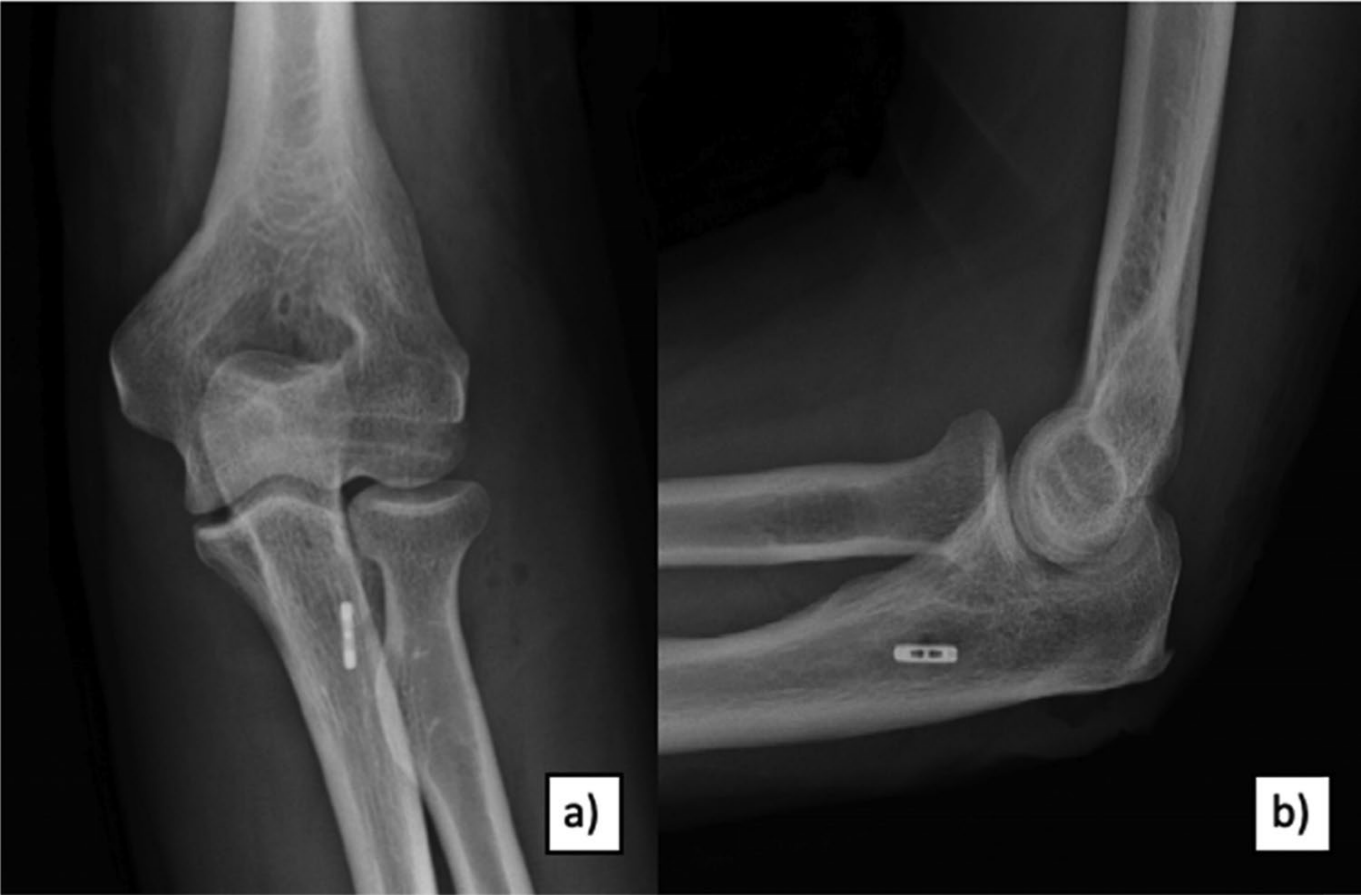
Postoperative X-rays **a** a.p. and **b** lateral after LUCL reconstruction using ipsilateral triceps graft: flip button fixation at the ulnar side and SwiveLock anchor (Arthrex Napels, FL, USA) fixation at the humeral condyle

Following elbow arthroscopy using the lateral decubitus position, LUCL reconstruction was performed according to Dehlinger et al. in all evaluated patients [15]. A triceps tendon autograft was harvested at the ipsilateral elbow in all cases via the extended Kocher approach. A titanium flip button was intramedullary placed for graft fixation at the ulnar supinator crest. For humeral restoration, a 5 mm drill hole was created to fix the graft with a 4.75 mm SwiveLock anchor (Arthrex Napels, FL, USA) (Fig. 3).

The extensor insertions were transosseously reattached. Three patients (*n* = 2 of group EPI, *n* = 1 of group TRAUMA) received additional ulnar nerve neurolysis because of irritations.

All patients were postoperatively immobilized for 2 days in a cast and a hinged brace was subsequently applied for 4 weeks. Physical therapy with free active motion was allowed from the first postoperative day.

### Follow-up evaluation

Physical examination consisted of ROM measurement and manual stability testing (pincer grip test, posterolateral rotatory drawer test, pivot shift test) [23–25]. For objective functional assessment, the Mayo elbow performance Score (MEPS) was applied. Additionally, PROs (patient rated outcomes) consisting of the visual analogue scale for pain (VAS), the disability of the arm, shoulder and hand questionnaire (DASH), the patient-rated elbow evaluation (PREE) and the subjective elbow evaluation (SEV) were recorded. Postoperative complications were retrospectively evaluated.

### Statistical analysis

An a priori power analysis was conducted using G*Power software [26]. Based on our clinical experience, an increase of 9 points for the MEPS was considered as clinically relevant. Based on previous data of Sanchez-Sotelo et al., a postoperative MEPS score of 85 points can be assumed for patients after LUCL reconstruction [27]. Combined with an expected mean standard deviation of 10 points, an effect size of 1.1 was calculated. Therefore, a total sample size of 28 patients was needed to achieve a statistical power of 0.8.

All calculations were performed with SPSS Statistics (Version 25, Property IBM Corp., NY, USA). Statistical means, minimum, maximum and standard deviations were calculated for continuous variables. The Mann–Whitney *U* test was used to compare group EPI with group TRAUMA as nonparametric test for the null hypothesis with a significance level of *p* < 0.05.

## Results

Preoperative and postoperative range-of-motion (ROM) was measured using a goniometer [28]. No statistical differences in ROM were found between the groups (Table 2).

**Table 2:** Pre- and postoperative ROM

	Group EPI (*n* = 17)	Group TRAUMA (*n* = 14)	*p* value
preROM flexion ± SD [°]	136.2 (± 6.3)	130.7 (± 15.4)	0.799
preROM extension ± SD [°]	− 0.9 (± 5.1)	2.5 (± 8.5)	0.296
postROM flexion ± SD [°]	137.9 (± 4.0)	138.6 (± 6.6)	0.296
postROM extension ± SD [°]	− 0.5 (± 3.4)	− 0.1 (± 5.8)	0.593

*preROM* preoperative range-of-motion, *postROM* postoperative range-of-motion

**Fig. 4:**
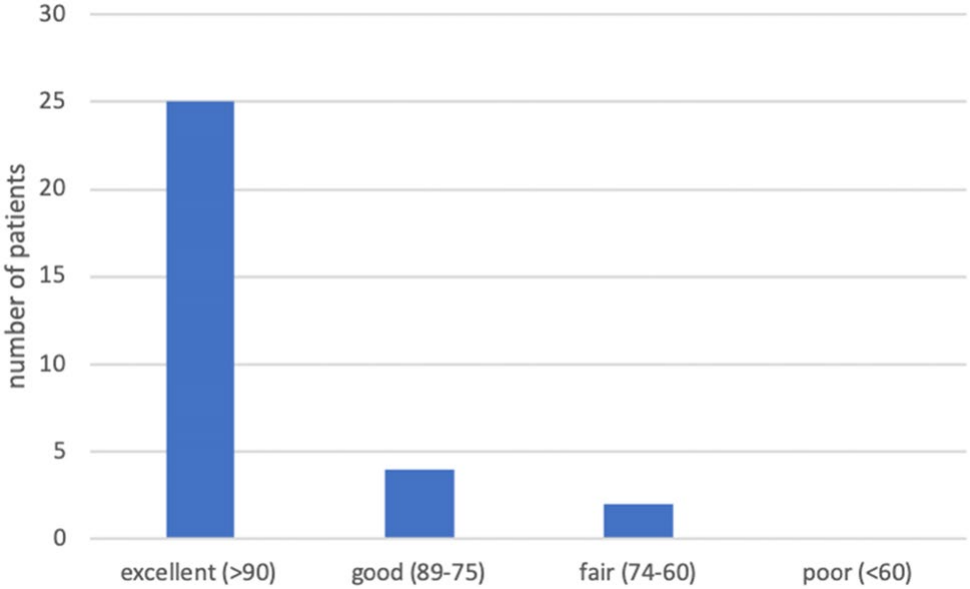
Rating system of the MEPS according to Nestor and Morrey [29]

The scoring outcomes are summarized in Table 3. Even though overall functional results of the MEPS were rated as good or excellent in 93.5% for both groups, patients in group EPI showed superior results (*p* = 0.034) (Fig. 4) [29]. Moreover, group EPI showed increased values for the VAS, the PREE, the DASH score and the SEV than group TRAUMA without statistical differences (Table 3).

**Table 3:** MEPS and PROs

	Group EPI (*p* = 17)	Group TRAUMA (*p* = 14)	*p* value
MEPS ± SD	98.9 (± 3.7)	91.1 (± 12.6)	0.034*
VAS ± SD	1 (± 1.8)	1.9 (± 3.2)	0.566
PREE ± SD	9.3 (± 15.7)	22.4 (± 26.1)	0.396
DASH ± SD	7.7 (± 11.8)	16.0 (± 19.4)	0.409
SEV ± SD	92.9 (± 8.3)	87.9(± 15.4)	0.632

Excellent: > 90 points in MEPS, good: 89–75 points in MEPS, fair: 74–60 points in MEPS, poor < 60 points in MEPS

*VAS* visual analogue scale for pain, *MEPS* Mayo elbow performance score, *PREE* patient rated elbow evaluation, *DASH* disabilities of the arm, shoulder and hand, *SD* standard deviation, *SEV* subjective elbow evaluation

*Statistically significant difference (*p* < 0.05)

Two out of 31 patients (6.5%; both of group TRAUMA) showed posterolateral re-instability during follow-up examination. Both patients had fracture dislocation injuries requiring prior surgeries. One of the two patients had no complaints and the other one denied reoperation. The re-instability rate was not statistically relevant comparing both groups (*p* > 0.05).

According to the Beighton score, general joint hyperlaxity did not differ significantly in both groups (*p* > 0.05) (Table 1) [30]. No statistical differences were found for steroid injections, previous surgeries, re-instability and revision surgeries regarding PROs for both groups (*p* > 0.05).

## Complications

In 12.9% of evaluated patients, a revision surgery was required due to different complications. In group EPI, one patient suffered from a deep infection with propioni acnes bacterium and had to undergo multiple debridement. The second patient showed persistent ulnar nerve symptoms and required neurolysis subsequently. In group TRAUMA, one patient also underwent secondary neurolysis of the ulnar nerve. The second patient received an arthroscopic arthrolysis due to elbow stiffness. Thus, the reoperation rate, therefore, was 11.8% in group EPI and 14.3% in group TRAUMA, respectively.

## Discussion

The most important finding of the present study was that the LUCL reconstruction procedure using a triceps tendon autograft, reliably restores posterolateral elbow stability, regardless of the initial pathogenesis. No significant differences with respect to the pathogenesis were seen in ROM, evaluated PROs and the recurrent instability rate. Nevertheless, a slight trend towards better PROs in patients with atraumatic PLRI was observed, however without reaching statistical significance. Solely the MEPS showed a statistically significant difference in favor of an atraumatic pathogenesis, while 93.5% of all evaluated patients obtained good to excellent results.

The present results of predominantly the MEPS are comparable to previously published data just in terms of the MEPS [2, 5, 14]. Jones et al. described a docking technique with palmaris autograft for reconstruction of PLRI in 8 patients; a MEPS of 87.5 was achieved 7.1 years postoperatively [2]. Olsen et al. reported a MEPS of 92 points 44 months after LUCL reconstruction with ipsilateral triceps graft in 18 patients [5]. In the present study, one possible explanation for the slight statistical difference in the MEPS of both groups could be due to the fact that both patients with re-instability were in the traumatic group, depreciating the groups’ mean MEPS through the scores’ stability section. Consequently, the clinical relevance remains debatable.

The underlying pathology of PLRI (traumatic vs. atraumatic LUCL insufficiency) has not yet been subject to detailed investigation. However, one study included both entities (atraumatic and traumatic pathologies) leading to PLRI in the elbow. Sanchez-Sotelo et al. reported of 32 and 12 patients after LUCL reconstruction with palmaris longus autograft and LUCL repair, respectively [27]. PLRI was caused by traumatic events, previous surgeries and described as unknown in 31, 7, and 4 patients, respectively. In contrast to the reported outcomes, Sanchez-Sotelo et al. reported superior results for patients with traumatic PLRI. However, the variety of several surgical techniques used in their study (repair vs. reconstruction) and different pathologies leading to small inhomogeneous comparative groups, renders the results open to interpretation. In the presented series, patients with traumatic PLRI tended to inferior PROs a greater re-instability rate without reaching statistical difference. We assumed that sequalae of the initial traumatic impact of, including posttraumatic osseous deformities and concomitant osteochondral lesions could lead to inferior subjective and objective results and ongoing impairment. For atraumatic reasons of PLRI, previous studies reported a correlation between chronic epicondylitis humeri radialis and steroid injections, leading to secondary insufficiency of the LUCL [10, 11, 13, 14]. Three cases were reported by Kalainov et al. [11], one case example by Chanlalit et al. [31], and 14 cases by Shim et al. [14]. Shim et al. reported significant improvement in functional outcomes and PROs following LUCL reconstruction with ipsilateral palmaris longus autograft. The MEPS and DASH score improved from preoperatively 60 to 91 postoperatively and from 48.0 to 13.5 points, respectively. The presented group of atraumatic patients (*n* = 14) in the current study achieved slightly better postoperative results in the MEPS with 98.9 ± 3.7, as well as for the DASH score 7.7 ± 11.8. However, preoperative values are not comparable due to missing data in the present study. Overall, recent literature shows good to excellent functional results at mid- to long-term follow-up for 85–90% after surgical restoration of posterolateral elbow stability, irrespective of initial pathology and the used surgical technique [2, 3, 14, 17, 27, 32].

In the present series, two patients indicated re-instability at follow-up survey. Interestingly, both belonged to the traumatic group (group TRAUMA) with accompanying fractures. Reasons for this skew may include altered osseous configuration and chondral lesions, which could lead to persisting micro-instability. The rate of re-instability in group TRAUMA was 14.3%, which is in accordance with the previously published re-instability rates after LUCL reconstruction after posttraumatic PLRI [18]. The aforementioned study by Sanchez-Sotelo reported a re-instability rate of 11% with two out of five patients receiving a revision stabilization procedure. Olsen and Sojbjerg evaluated a series of 18 patients with posttraumatic PLRI and LUCL reconstruction using an ipsilateral triceps tendon autograft [5]. The re-instability rate was 22% in the clinical testing, but only one patient had subjective complaints and underwent reoperation. These findings resemble the results of our study. The re-instability rate following LUCL reconstruction in atraumatic PLRI is rather high with 21.4% [14]. However, Shim et al. also argue that re-stabilization procedures were not necessary due to only mild symptoms [14].

In a systematic review including 8 studies, with 130 patients, the re-instability rate was 8% and the overall complication rate 11% following LUCL reconstruction [18]. These complication and revision rates are comparable to the present study with 12.9% for both groups. Reasons for reoperation were neuritis of the ulnar nerve, deep wound infection and arthrofibrosis.

The main weakness of this study is its retrospective design with a limited sample size. The number of evaluated subjects, however, is comparable to previous studies and a reasonable follow-up rate of 86.1% with a midterm follow-up time was achieved [2, 5, 14, 27, 32, 33]. Furthermore, the study contains distinguishing strengths. First, PLRI was confirmed under direct visualization by arthroscopic testing in all patients. Second, all LUCL reconstruction procedures were performed in a standardized uniform fashion identically (ipsilateral triceps tendon autograft, hybrid fixation technique) minimizing performance bias. Third, a priori power analysis was conducted.

## Conclusion

LUCL reconstruction using a triceps tendon autograft for the treatment of PLRI leads to good to excellent clinical outcomes regardless of the underlying pathogenesis (traumatic vs. atraumatic) with a low re-instability rate (6.5%). However, the propensity for re-instability is higher for traumatic PLRI. Moreover, traumatic PLRI tends to result in inferior PROs and clinical outcomes.
